# Ameliorating Fibrotic Phenotypes of Keloid Dermal Fibroblasts through an Epidermal Growth Factor-Mediated Extracellular Matrix Remodeling

**DOI:** 10.3390/ijms22042198

**Published:** 2021-02-23

**Authors:** Hyunbum Kim, Laurensia Danis Anggradita, Sun-Jae Lee, Sung Sik Hur, Joonsuk Bae, Nathaniel Suk-Yeon Hwang, Seung Min Nam, Yongsung Hwang

**Affiliations:** 1 Soonchunhyang Institute of Medi-Bio Science (SIMS), Soonchunhyang University, Cheonan-si 31151, Korea; tiggerhy@snu.ac.kr (H.K.); laurensiadanis@sch.ac.kr (L.D.A.); sstahur@sch.ac.kr (S.S.H.); 2 School of Chemical and Biological Engineering, Institute of Chemical Processes, Seoul National University, Seoul 08826, Korea; nshwang@snu.ac.kr; 3 Department of Integrated Biomedical Science, Soonchunhyang University, Asan-si 31538, Korea; 4 Department of Plastic and Reconstructive Surgery, Soonchunhyang University Bucheon Hospital, Soonchunhyang University College of Medicine, Bucheon-si 14584, Korea; rokitanski@naver.com (S.-J.L.); 132663@schmc.ac.kr (J.B.)

**Keywords:** keloid scar, hypertrophic scar, dermal fibroblast, epidermal growth factor, ECM remodeling, traction force

## Abstract

Keloid and hypertrophic scars are skin fibrosis-associated disorders that exhibit an uncontrollable proliferation of fibroblasts and their subsequent contribution to the excessive accumulation of extracellular matrix (ECM) in the dermis. In this study, to elucidate the underlying mechanisms, we investigated the pivotal roles of epidermal growth factor (EGF) in modulating fibrotic phenotypes of keloid and hypertrophic dermal fibroblasts. Our initial findings revealed the molecular signatures of keloid dermal fibroblasts and showed the highest degree of skin fibrosis markers, ECM remodeling, anabolic collagen-cross-linking enzymes, such as lysyl oxidase (LOX) and four LOX-like family enzymes, migration ability, and cell–matrix traction force, at cell–matrix interfaces. Furthermore, we observed significant EGF-mediated downregulation of anabolic collagen-cross-linking enzymes, resulting in amelioration of fibrotic phenotypes and a decrease in cell motility measured according to the cell–matrix traction force. These findings offer insight into the important roles of EGF-mediated cell–matrix interactions at the cell–matrix interface, as well as ECM remodeling. Furthermore, the results suggest their contribution to the reduction of fibrotic phenotypes in keloid dermal fibroblasts, which could lead to the development of therapeutic modalities to prevent or reduce scar tissue formation.

## 1. Introduction

Keloid and hypertrophic scars are defined as abnormal healing of injured or irritated skin during wound healing caused by a pathologically uncontrollable proliferation of fibroblasts in the dermis layer, which eventually results in overabundant accumulation of extracellular matrix (ECM) components [1,2,3,4]. Keloid scars form enlarged and extended scar tissues beyond the original wound margins and often recur after surgical excision, whereas hypertrophic scars do not extend beyond the wound boundary [5,6]. Although the biochemical and biophysical properties of keloid and hypertrophic scars are distinguishable, the pathophysiology of keloid formation and classification remain incompletely identified [7]. Furthermore, many treatment methods for keloids have been introduced, including intraregional steroid injection, pressure garments, cryosurgery, laser therapy, and radiotherapy. Nevertheless, effective treatment of keloid scars with good clinical outcomes and high efficacy has been a daunting challenge in recent decades [8,9].

Transforming growth factor (TGF)-β1 signaling is a major pathway involved in keloid pathogenesis, with TGF-β1 representing an essential fibrotic cytokine involved in skin fibrosis and ECM remodeling [10,11]. Among various skin ECMs, collagens are the most abundant fibrillar proteins, and their proper reorganization and remodeling are crucial for regulating various resident cells within native skin tissues during wound-healing processes [12]. Collagens are synthesized by activated myofibroblasts, with their synthesis and degradation orchestrated by various enzymes, including several catabolic matrix metalloproteinases (MMPs) for degradation of collagens as well as anabolic lysyl oxidase (LOX) and four lysyl oxidase-like (LOXL) family members that covalently form cross-links between collagens [13]. Thus, emerging evidence reveals that the LOX and LOXL family are involved in various diseases related to pathogenic tissue fibrosis, including idiopathic pulmonary fibrosis (IPF), renal fibrosis, cardiac fibrosis, hepatic fibrosis and systemic sclerosis [14,15,16,17,18]. However, their potential implications in keloid skin disorders are not fully understood. The epidermal growth factor (EGF) signaling pathway enhances fibroblast proliferation and the migration of vascular endothelial cells, as well as modulating the TGF-β1 signaling pathway [19,20]. A previous study reported that EGF treatment of human dermal fibroblasts downregulates ECM production, including expression of type I procollagen protein, and upregulates MMP-1 expression [21].

Numerous reports have demonstrated the potential application of traction force microscopy (TFM) for discovering the important roles of cell–matrix/cell–cell interactions at cell–matrix interfaces in regulating various cellular behaviors, including cell adhesion, proliferation, differentiation, disease progression and tissue formation [22,23,24]. There have been limited studies demonstrating the important aspects of pathophysiological imbalance between proteolytic enzyme-mediated ECM degradation and enzyme-associated ECM synthesis via collagen-cross-linking in keloid skin disorder. Therefore, in this study, we investigated whether exogenous EGF could ameliorate the fibrotic phenotypes of keloid and hypertrophic scar-derived dermal fibroblasts through modulation of ECM remodeling. Additionally, given the versatile capacity of TFM analysis to assess the cell motility of dermal fibroblasts, we employed fibronectin-conjugated polyacrylamide (PAA) hydrogels, with a normal skin-like Young’s modulus of 10.6 kPa, to measure cell–matrix traction stress in the presence of EGF in order to evaluate EGF-mediated changes in dermal fibroblast migration at cell–matrix interfaces.

## 2. Results

### 2.1. Histologic Evaluation of Normal, Hypertrophic, and Keloid (NHK) Scar Tissues

**Figure 1:**
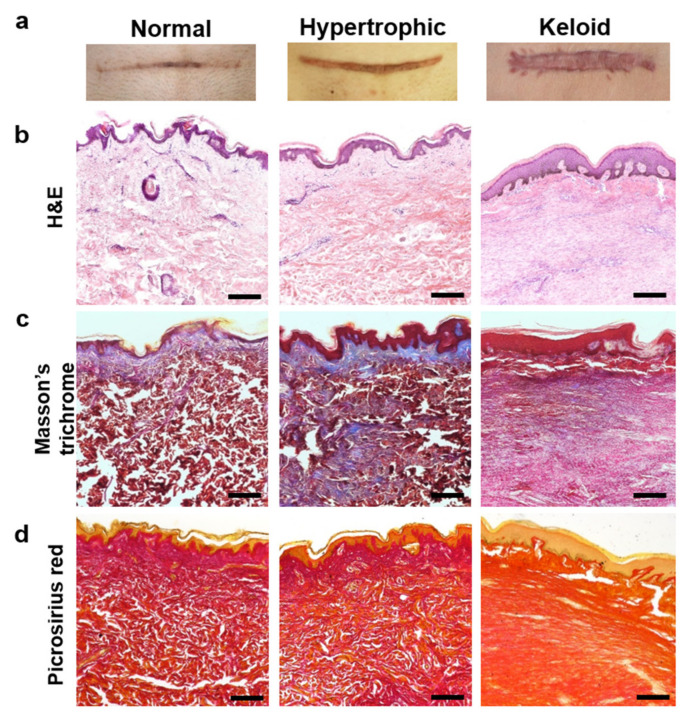
Histologic evaluation of skin tissues from patients with NHK scars. (**a**) Representative gross images of patient scar tissues. (**b**) Hematoxylin and eosin (H&E)-stained scar tissues for nuclei (purple) and deposited extracellular matrix (ECM, pink). (**c**) Masson’s trichrome-stained scar tissues for collagen deposition (blue) and cytoplasm (pink). (**d**) Picrosirius red-stained scar tissues for collagen deposition (red) and cytoplasm (orange). Scale bar = 200 µm.

Skin tissues were categorized as NHK scar tissues based on the Vancouver Scar Scale (VSS) (Table 1) by trained clinicians and pathologists. Age, gender and location of NHK scars were confirmed in each patient and sorted accordingly. VSS scores were determined by analyzing scales of vascularity, pigmentation, pliability and height of skin tissues (Figure 1a). The mean total VSS scores increased significantly in keloid and hypertrophic scars (10.00 ± 2.08 and 6.29 ± 1.704, respectively) as compared with baseline (normal skin tissue) scores. Histologic analyses to evaluate differences in dermal tissue revealed that both keloid and hypertrophic scars showed thickening of the epidermis, and more nuclei were observed in their dermis regions, which was confirmed by hematoxylin and eosin (H&E) staining (Figure 1b). To further evaluate fibrotic tissue formation in the dermis, Masson’s trichrome and Picrosirius red staining revealed abundant and dense collagen deposition in both keloid and hypertrophic scar tissues (Figure 1c,d). These results clearly suggest that hypertrophic and keloid scar tissues exhibit excessive ECM deposition on skin tissues, which is a typical phenotype of skin fibrosis pathology. Additionally, when trained clinicians and pathologists evaluated VSS scores for male and female patients in the hypertrophic scar group, no significant differences were observed in morphological characteristics of their scars. This is in agreement with previously reported studies where ethnic and genetic backgrounds, rather than gender, were primary factors determining the phenotypes of keloid and hypertrophic scars [25,26].

### 2.2. Isolation of Primary Dermal Fibroblasts from NHK Skin Tissues and Their Phenotypic Characterization

**Figure 2:**
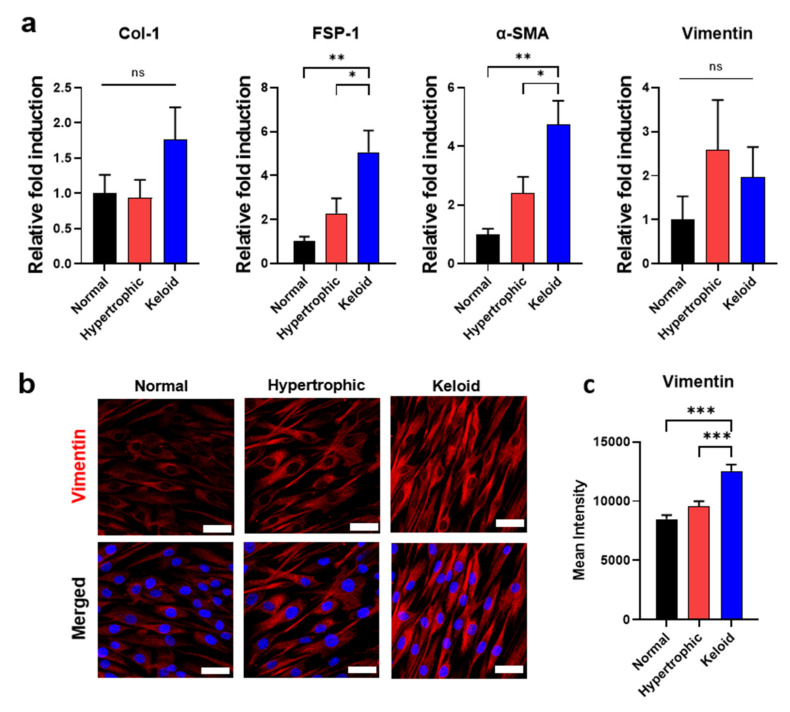
Isolation of dermal fibroblasts from NHK patient-derived skin tissues and their phenotypic characterization. (**a**) quantitative polymerase chain reaction (qPCR) of skin fibrosis and activation of myofibroblast-associated markers. (**b**) Immunofluorescence staining for vimentin (red) and nuclei (blue). Scale bar = 50 µm. (**c**) Mean intensity values of vimentin protein expression quantified by MATLAB. Data represent the mean ± SEM. * *p* < 0.05, ** *p* < 0.01, *** *p* < 0.001.

From the categorized human-patient-derived skin tissues, primary dermal fibroblasts were isolated and cultured in vitro. Our initial observation revealed that dermal fibroblasts isolated from HNK scar tissues showed similar typical spindle-cell morphology (Figure S1). To assess the fibrotic phenotypes of HNK dermal fibroblasts, we evaluated the expression of collagen type I (*Col-I*), fibroblast-specific protein-1 (*FSP-1*), α-smooth muscle actin (*α-SMA*), and vimentin, which were previously reported as skin fibrosis-/myofibroblast activation-associated markers [27,28]. As shown in Figure 2a, quantitative polymerase chain reaction (qPCR) results confirmed that keloid dermal fibroblasts showed significantly increased skin fibrosis activation of myofibroblast-associated gene expression as compared with the other two dermal fibroblast groups, which agreed with previously reported findings [29,30]. To confirm the gene expression profiles, we assessed protein expression of vimentin, a marker associated with skin fibrosis and myofibroblast activation [31,32,33]. Keloid dermal fibroblasts showed significantly higher expression levels of vimentin relative to the other two dermal fibroblast groups, with these results also confirmed by quantitative image analysis of immunofluorescence staining of vimentin (Figure 2b,c and Figure S3). Moreover, evaluation of gene expression up to the 7th passage for each group validated the stability of their fibrotic phenotype during multiple passages (Figure S2).

**Figure 3:**
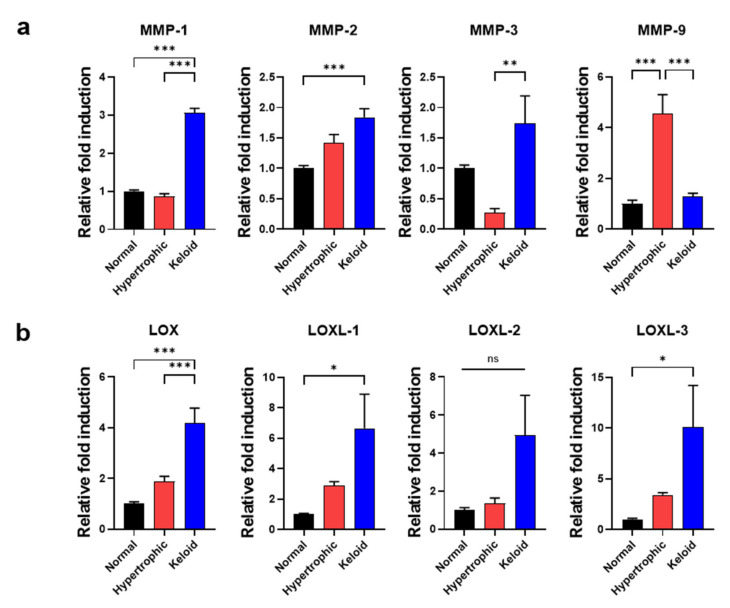
qPCR analysis of genes responsible for ECM remodeling and expressed in primary dermal fibroblasts from NHK patient-derived scar tissues. qPCR of (**a**) collagen-degrading *MMPs* and (**b**) collagen-cross-linking *LOX* and *LOXLs*. Data represented the mean ± SEM. * *p* < 0.05; ** *p* < 0.01; *** *p* < 0.001.

Given the limited number of reports demonstrating the relationship between hypertrophic and keloid scar-tissue formation and corresponding ECM remodeling, including activity by both catabolic and anabolic enzymes, we compared the gene-expression levels of *MMPs, LOX,* and *LOXLs,* which regulate ECM degradation and collagen cross-linking with various tissue origins [13], among NHK scar tissues. The results demonstrated that keloid dermal fibroblasts exhibited significantly increased expression of *MMP-1, MMP-2*, and *MMP-3* relative to normal or hypertrophic dermal fibroblasts (Figure 3a). However, *MMP-9* was significantly upregulated in only fibroblasts from hypertrophic scars. In addition to collagen-degrading enzymes, we examined the gene-expression levels of collagen-cross-linking enzymes, such as *LOX, LOXL-1, LOXL-2* and *LOXL-3*, finding that they were substantially upregulated in keloid dermal fibroblasts relative to either normal or hypertrophic dermal fibroblasts (Figure 3b). qPCR results and immunofluorescence staining of vimentin clearly suggested that keloid dermal fibroblasts exhibit fibrotic phenotypes of skin fibrosis and activation of myofibroblasts. Furthermore, ECM-remodeling enzymes, including both collagen-degrading and collagen-cross-linking enzymes, likely play an important role in the fibrotic phenotypes of keloid dermal fibroblasts.

### 2.3. Cell–Matrix Interaction-Mediated Increases in the Cell Motility of Keloid Dermal Fibroblasts

**Figure 4:**
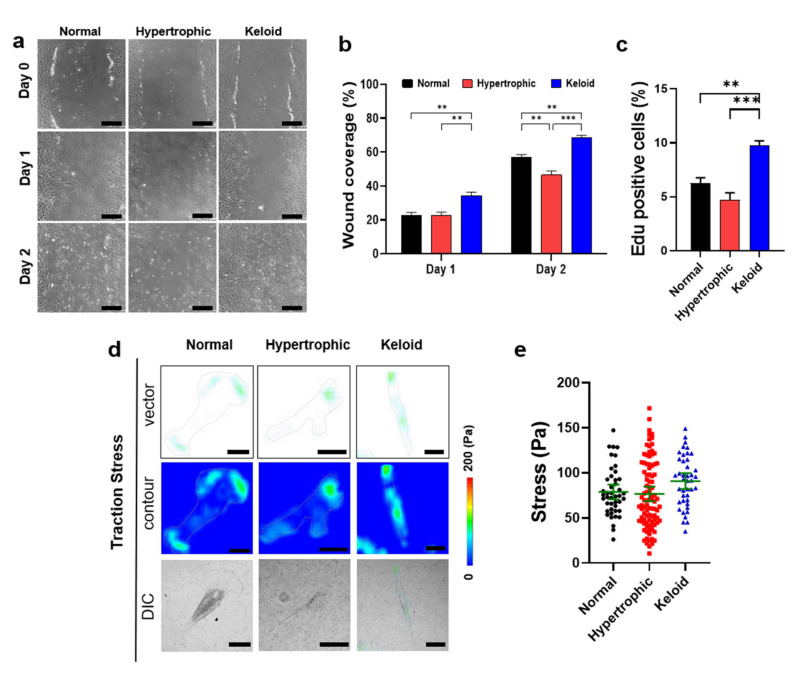
Characterization of the cell motility of dermal fibroblasts from NHK patient-derived scar tissues. (**a**,**b**) Wound-scratch assay for NHK dermal fibroblasts. Scale bar = 500 µm. Data represent the mean ± SEM. ** *p* < 0.01; *** *p* < 0.001. (**c**) Flow cytometry analysis of 5-ethynyl-2′-deoxyuridine (EdU)-positive cells. Data represent the mean ± SEM. ** *p* < 0.01; *** *p* < 0.001. (**d**,**e**) Traction stress data in normal (*n* = 44), hypertrophic (*n* = 96), and keloid dermal fibroblasts (*n* = 42). Scale bar = 50 µm. Data represent the mean (green line) ± 95% confidence interval (green error bars).

Because keloid dermal fibroblasts exhibit increased cell migration and proliferation abilities during multistage wound-healing processes [34], we examined the motility of primary dermal fibroblasts isolated from NHK scar tissues using a scratch-wound assay. Dermal fibroblasts were cultured until reaching confluence, and a wound scratch was created with a 1-mL pipette tip. We observed a significantly higher degree of cell motility in keloid dermal fibroblasts in 48 h, which agreed with a previous report [34], whereas hypertrophic dermal fibroblasts showed the lowest migratory potential (Figure 4a,b). Additionally, to evaluate the proliferative potential of all dermal fibroblasts, we performed a 5-ethynyl-2′-deoxyuridine (EdU) assay using flow cytometry and immunofluorescence analyses (Figure 4c and Figure S4). Concordant with the migration ability of keloid dermal fibroblasts, the results clearly demonstrated that keloid dermal fibroblasts showed significantly elevated proliferation, whereas hypertrophic dermal fibroblasts exhibited the least proliferative capacity relative to normal dermal fibroblasts.

We then examined differences in cell–matrix traction forces in order to determine the cell-motility behaviors among dermal fibroblasts isolated from NHK scar tissues. To evaluate cell-motility behaviors at the cell–matrix interface, we measured the cell–matrix traction force using fibronectin-conjugated PAA hydrogels with a Young’s modulus of 10.6 kPa (equivalent to that of native skin tissue) [30,35]. TFM analyses allowed visualization of traction stress vector (top) and displacement contour maps (middle) of traction stress, where the arrowheads indicate the directionality of traction stress exerted by adhered dermal fibroblasts onto PAA hydrogels (Figure 4d,e). The results revealed that keloid dermal fibroblasts exerted the highest traction stresses, although they were not statistically significant. Thus, these findings suggest that a higher degree of traction forces exerted by keloid dermal fibroblasts might influence a higher degree of migration capacity.

### 2.4. Exogenous EGF-Mediated Amelioration of Fibrotic Phenotypes of Keloid Dermal Fibroblasts through Modulation of ECM Remodeling

**Figure 5:**
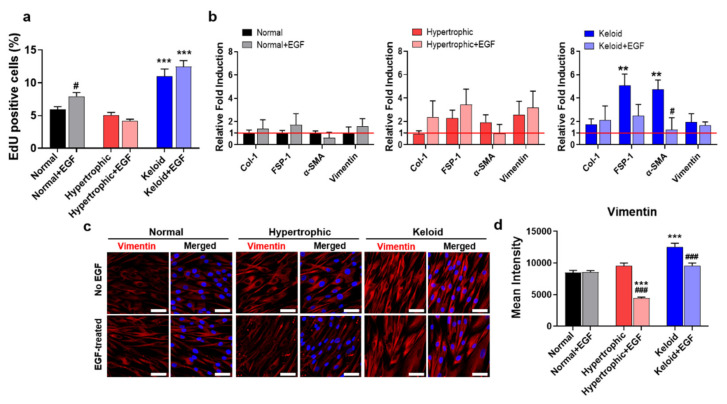
Exogenous EGF-mediated amelioration of the fibrotic phenotypes of keloid dermal fibroblasts. (**a**) Flow cytometry analysis of EdU-positive cells. (**b**) qPCR of skin fibrosis-/activation of myofibroblast-associated markers in the presence or absence of EGF treatment. Data were normalized against those of normal fibroblasts without EGF treatment. The red solid line represents gene expression in the control group (normal dermal fibroblasts). Data represent the mean ± SEM. ** *p* < 0.01, *** *p* < 0.001 vs. normal fibroblasts without EGF treatment; and # *p* < 0.05, vs. each control. (**c**) Immunofluorescence staining of vimentin (red) and nuclei (blue). Scale bar = 50 µm. (**d**) Mean intensity values of vimentin protein expression quantified by MATLAB. Data represent the mean ± SEM. *** *p* < 0.001 vs. normal fibroblasts without EGF treatment; and ### *p* < 0.001 vs. each control.

EGF plays a critical role in regulating various cellular functions of dermal fibroblasts, thereby influencing skin homeostasis and wound-healing cascades [36,37]. To characterize the distinctive nature of dermal fibroblasts isolated from different pathological conditions, such as NHK scar tissues, we investigated the roles of exogenous EGF in the fibrotic phenotypes of these dermal fibroblasts in terms of their gene and protein expression. As shown in Figure 5a and Figure S6, treatment of cells with exogenous EGF significantly increased the proliferating cell populations of both normal and keloid dermal fibroblasts, whereas hypertrophic dermal fibroblasts exhibited slightly decreased proliferative capacity. Additionally, treatment of the dermal fibroblasts with EGF resulted in no significant difference in cell size, although the cells acquired a spindle-shaped morphology, which was evident according to decreased cell circularity (Figure S5).

To investigate the effects of EGF treatment on the cellular functions of dermal fibroblasts, we examined gene expression of skin fibrosis-/activation of myofibroblast-specific markers (Figure 5b). Although EGF-treated normal and hypertrophic dermal fibroblasts showed increased gene expression levels of *Col-I* and *FSP-1*, *α-SMA* expression decreased slightly (*p* > 0.05). Additionally, culture of keloid dermal fibroblasts with EGF resulted in decreased expression of *FSP-1, α-SMA,* and vimentin. To confirm the qPCR results, we evaluated vimentin protein levels following EGF treatment with immunofluorescence staining, demonstrating amelioration of the fibrotic phenotypes of dermal fibroblasts from hypertrophic and keloid scar tissues, whereas no difference was observed in normal fibroblasts (Figure 5c,d and Figure S7).

**Figure 6:**
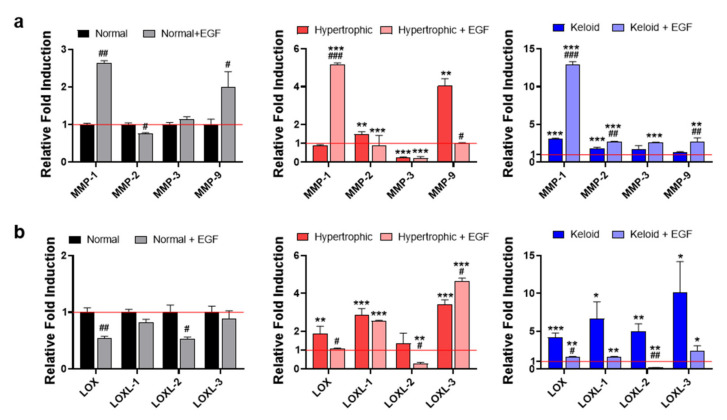
Exogenous EGF-mediated ECM remodeling in dermal fibroblasts from NHK patient-derived scar tissues. qPCR of (**a**) collagen-degrading *MMPs* and (**b**) collagen-cross-linking *LOX* and *LOXLs* in the presence or absence of EGF. Data were normalized against normal fibroblasts without EGF treatment. The red solid line represents gene expression in the control group (normal dermal fibroblasts). Data represent the mean ± SEM. * *p* < 0.05, ** *p* < 0.01, *** *p* < 0.001 vs. normal fibroblasts without EGF treatment; and # *p* < 0.05, ## *p* < 0.01, ### *p* < 0.001 vs. untreated controls.

Furthermore, to investigate how exogenous EGF ameliorates the fibrotic phenotypes of keloid dermal fibroblasts, we evaluated subsequent EGF-mediated changes in ECM remodeling, including the activities of both catabolic and anabolic enzymes. As previously reported [21], EGF treatment of all dermal fibroblasts significantly induced an increase in *MMP-1* expression, and both normal and keloid dermal fibroblasts showed increased *MMP-9* expression, whereas hypertrophic dermal fibroblasts showed a significant decrease in *MMP-9* expression (Figure 6a). Additionally, keloid dermal fibroblasts exhibited increased *MMP-2* and *MMP-3* expression, whereas no differences in expression were observed in normal and hypertrophic dermal fibroblasts. Interestingly, keloid dermal fibroblasts showed a substantial EGF-mediated decrease in *LOX* and *LOXL* expression relative to normal and hypertrophic dermal fibroblasts, whereas normal and hypertrophic dermal fibroblasts showed a significant decrease in *LOXL-2* compared with cells not receiving EGF treatment (Figure 6b). These results indicated that EGF treatment ameliorated the fibrotic phenotypes of keloid dermal fibroblasts by modulating ECM-remodeling processes.

### 2.5. Exogenous EGF-Mediated Changes in Cell-Matrix Traction Force

**Figure 7:**
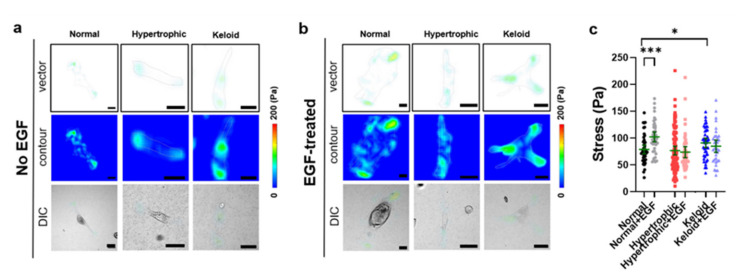
Characterization of the cell motility of dermal fibroblasts from NHK patient-derived scar tissues following EGF treatment. (**a**) Traction stress data from normal (*n* = 44), hypertrophic (*n* = 96), and keloid dermal fibroblasts (*n* = 42) without EGF treatment. (**b**) Traction stress data in normal (*n* = 42), hypertrophic (*n* = 46), and keloid dermal fibroblasts (*n* = 34) with EGF treatment. Scale bar = 50 µm. Data represent the mean (green line) ±95% confidence interval (green error bars). (**c**) Traction stress calculated using normal (*n* = 44), hypertrophic (*n* = 96), and keloid dermal fibroblasts (*n* = 42). Data represent the mean (green line) ± 95% confidence interval (green error bars). * *p* < 0.05; *** *p* < 0.001 vs. normal fibroblasts without EGF treatment.

To assess whether EGF-mediated cell-adhesion affects the mobility of all dermal fibroblasts isolated from NHK scar tissues, we compared changes in their traction stress in terms of cell–matrix traction force using TFM. TFM analyses demonstrated that normal dermal fibroblasts showed an EGF-mediated increase in traction stress, whereas both hypertrophy and keloid dermal fibroblasts exhibited EGF-mediated decreases (Figure 7). Moreover, distribution of the cell–matrix traction force became well-distributed throughout the cells. These findings demonstrated the important roles of EGF on the cell–matrix traction force and its possible effect on the migratory ability of dermal fibroblasts and their contribution to skin fibrosis and myofibroblast activation.

## 3. Discussion

Although there have been numerous attempts to determine the molecular mechanism underlying skin fibrosis and myofibroblast activation in keloid skin disorders, little is known about keloid pathophysiology [1,3,4,6]. In this study, we characterized and compared the gene and protein expression profiles of scar tissue-derived fibroblasts isolated from NHK scar tissues. The initial findings provided strong evidence of distinct differences between normal versus hypertrophic and keloid scar tissues in terms of fibrotic ECM deposition at both the tissue and cellular levels. Such differences may be the consequence of aberrant ECM remodeling driven by imbalances between collagen-degrading catabolic enzymes and their collagen-cross-linking anabolic counterparts.

Despite similarities between hypertrophic and keloid scar-derived dermal fibroblasts, which exhibited an elevated expression of skin fibrosis-/myofibroblast activation-associated markers (Figure 2) relative to normal scar-derived dermal fibroblasts, keloid dermal fibroblasts showed a higher degree of gene expression of collagen-cross-linking anabolic enzymes (Figure 3), in agreement with previous studies [38]. Similar to these results, previous studies also reported the overexpression of LOX and LOXL-2 in various pathological conditions characterized by fibrotic phenotypes, including IPF, renal fibrosis, cardiac fibrosis, skin aging and systemic sclerosis. Moreover, inhibition of LOX and LOXL-2 expression reduced fibrosis in animal models [14,15,16,17,18]. These findings indicated that LOX and LOX-like family members can serve as potential therapeutic targets in skin fibrosis and keloid scar tissue formation.

Additionally, it has been extensively speculated that MMPs, as major ECM proteases, can play a pivotal role in collagen degradation and ECM remodeling during wound healing as well as scar tissue formation [39]. Among various MMPs overexpressed in keloid scars, MMP-1, an interstitial collagenase primarily secreted by keratinocytes, plays an essential role in disrupting the collagen from its triple-helix structure and loosening cell-matrix adhesions within a wound matrix, thus promoting the re-epithelialization process [13]. MMP-2 and MMP-9 are gelatinase proteins that degrade gelatin and remove the abnormal or unfolded collagen that previously has been cleaved by collagenase [40]. More importantly, while these MMP-1/2/3 can promote migration of numerous cell types within skin tissue, such as fibroblasts, keratinocytes, smooth muscle cells, endothelial cells and macrophages, they are particularly elevated in keloid scar-derived fibroblasts, triggering abnormal ECM degradation which then leads to excessive ECM deposition [41,42]. MMP-3 is a stromelysin protein that cleaves not only collagen but also non-collagenous molecules such as proteoglycan, laminin and fibronectin, thus influencing wound contraction [43].

We then investigated whether EGF treatment could reduce the fibrotic phenotypes of hypertrophic and keloid dermal fibroblasts via creating alterations in ECM remodeling. Previously, numerous studies demonstrated the important role of the TGF-β1 signaling pathway in keloid pathophysiology [10,11]. More specifically, it was reported that TGF-β1 promotes myofibroblast activation by inducing the overexpression of α-SMA, MMP-2, and MMP-9, while inhibiting MMP-1 expression, which results in excessive collagen accumulation [12,44]. On the other hand, the EGF signaling pathway has been shown to improve cell motility, dermal fibroblast proliferation and wound healing [19,21]. Interestingly, our results indicated that exogenous EGF alleviated the fibrotic phenotype only in keloid dermal fibroblasts, evident by a significantly decreased gene expression of FSP-1, α-SMA and vimentin, as well as decreased vimentin protein expression (Figure 5). This could be achieved through modulation of LOX, LOXLs and MMP-1 expression (Figure 6). The findings were also consistent with previous studies demonstrating the potential antifibrotic effect of exogenous EGF on various in vivo fibrosis models, including liver, heart and skeletal muscle models, where the supplemented EGF upregulated MMP-1 expression and subsequently alleviated the fibrotic phenotypes [21,45,46,47]. Although dermal fibroblasts respond to exogenous EGF, the degree of their responsiveness differs significantly based on their cellular phenotypes, such as aging and pathological conditions [36,37,48]. Therefore, these findings suggest that the keloid dermal fibroblasts in our study exhibited reduced fibrotic phenotypes owing to decreased expression of LOX and LOXLs as well as significantly increased expression of MMP-1 with its higher sensitivity to EGF.

In addition to EGF-mediated ECM remodeling, we assessed the contribution of EGF to the cell–matrix interaction of dermal fibroblasts. Although emerging evidence has revealed the critical roles of focal adhesion-associated cell–matrix interactions in cell adhesion and migration across various cell types [23,49,50], relatively less is known about the role of EGF in regulating hypertrophy and keloid dermal fibroblasts via the cell–matrix traction force. Here, TFM analyses indicated a strong correlation between cell–matrix traction stress and cell motility among various dermal fibroblasts. Additionally, culture of dermal fibroblasts on the normal skin-matching stiffness of the cell–adhesive matrix revealed a proportional degree of cell motility to the cell–matrix traction stress exerted by adhered dermal fibroblasts, with keloid dermal fibroblasts showing the highest cell–matrix traction stress (Figure 4 and Figure 7). However, treatment of keloid dermal fibroblasts with EGF decreased their cell–matrix traction stress. Similarly, recent advancements in the field of cell mechanics have enabled the observation that keloid dermal fibroblasts exhibit a greater magnitude of force generation than normal dermal fibroblasts through focal adhesion complexes during cell migration, as determined by atomic force microscopy measurements [51]. Therefore, these TFM results suggest a correlation between the migration capacity of keloid dermal fibroblasts and its contribution to fibrotic phenotypes and myofibroblast activation. Moreover, the current findings highlight the potentially important role of cell–matrix interactions of keloid dermal fibroblasts within their microenvironment during in vivo wound healing and excess ECM production. Although characteristic differences were noted between NHK dermal fibroblasts, the principal mechanism underlying the development of keloid skin disorders remains unidentified. Nevertheless, our observations strengthen the current understanding of keloid pathophysiology and establish a basis for discovering effective therapeutic strategies to prevent and treat scar tissue formation.

In summary, this study characterized dermal fibroblasts within hypertrophic and keloid scar tissues in relation to ECM remodeling. Furthermore, EGF ameliorated the fibrotic phenotypes of keloid dermal fibroblasts by modulating collagen degradation and synthesis. In addition, the results of the cell–matrix traction force analysis indicated that this analytical method can effectively distinguish various cellular functions of dermal fibroblasts isolated from NHK scars, thus providing a reliable platform for the evaluation of subtle differences in cell–matrix interactions associated with the progression of skin-disease pathology.

## 4. Materials and Methods

### 4.1. Patients and Sample Collection

After obtaining written informed consent from all patients according to a protocol approved by the Institutional Review Board of Soonchunhyang University Bucheon Hospital (SCHBC_IRB_2017-08-010), skin tissues, including NHK scar tissues, from the central dermal layer of 20 patients (Table 1) were obtained for fibroblast isolation and immunohistochemistry. For normal skin tissues, samples were obtained from tissue excision during breast reconstruction using a *Latissimus dorsi* musculocutaneous flap. All experiments involving human subjects were conformed to the Declaration of Helsinki.

### 4.2. Isolation of Dermal Fibroblasts and In Vitro Cell Culture

Before enzymatic digestion of tissues, tissue fragments were placed in 15-mL conical tubes and washed with 10 mL of sterile phosphate-buffered saline (PBS) containing 1% penicillin-streptomycin (P/S; 10,000 U/mL of penicillin and 10,000 g/mL of streptomycin; Gibco-BRL, Gaithersburg, MD, USA) six times under vigorous agitation, with the tubes changed between each wash. Tissue fragments were then transferred to Petri dishes and chopped into small pieces (~1 mm^3^ in size) under sterile conditions. Tissue pieces were digested with serum-free high-glucose Dulbecco’s modified Eagle medium (DMEM; Gibco-BRL) containing 1% P/S and 1 mg/mL collagenase type I for 2 h at 37 °C in a shaking incubator. To stop the enzymatic digestion, an equal volume of DMEM containing 10% fetal bovine serum (FBS; Gibco-BRL) was added, and the cell suspension was passed through a 100-μm cell strainer (BD Falcon; BD Biosciences, Franklin Lakes, NJ, USA) to remove undigested tissue and cell aggregates. The filtered cells were washed twice with serum-free DMEM and seeded into 12-well plates at a seeding density of 5 × 10^3^ cells/cm^2^. The cells were incubated in a humidified incubator with a 5% CO_2_ atmosphere at 37 °C in growth medium containing DMEM supplemented with 10% FBS, 1% L-glutamine (200 mM; Gibco-BRL), and 1% P/S.

### 4.3. H&E and Masson’s Trichrome Staining

Tissue specimens, which were previously fixed in 4% paraformaldehyde (PFA) for 24 h, were dehydrated using an ethanol series and embedded in paraffin. The paraffin-embedded tissues were sectioned into 10-µm-thick sections, which were stained with H&E and Masson’s trichrome for histopathologic evaluation. Tissue samples were first stained for nuclei with hematoxylin (Mayer’s modified; Abcam, Cambridge, UK) for 3 min and rinsed several times with an excess amount of water, followed by dipping several times in 1% (*w*/*v*) acid ethanol for destaining. Specimens were then stained with eosin (Sigma-Aldrich, St. Louis, MO, USA) for 20 s, dehydrated in a graded ethanol series, and treated with xylene prior to mounting. For Masson’s trichrome staining, the specimens were stained with iron hematoxylin and Biebrich Scarlet-Acid Fuchsin solution according to manufacturer’s instructions (Polyscience, Niles, IL, USA). All specimens were analyzed using an inverted microscope (Eclipse Ti-U; Nikon, Tokyo, Japan) at the Soonchunhyang Biomedical Research Core-Facility of the Korea Basic Science Institute (KBSI).

### 4.4. qPCR

Total RNA was extracted using Trizol reagent (Invitrogen, Carlsbad, CA, USA), and reverse transcription was performed using ReverTra Ace qPCR RT master mix with gDNA Remover (Toyobo, Osaka, Japan) according to manufacturer’s instructions. Briefly, for cDNA synthesis, 1 µg of RNA was used as a template in a 10-µL reaction, and qPCR was performed using SYBR Green real-time PCR master mix (Toyobo) on a StepOnePlus real-time PCR system (Applied Biosystems, Foster City, CA, USA) at the Soonchunhyang Biomedical Research Core-Facility of KBSI. All experiments were performed with at least three to seven biological replicates for each group, and the expression levels of genes of interest were normalized against glyceraldehyde-3-phosphate dehydrogenase (GAPDH). The ΔCt values were determined as follows: Ct^target^ − Ct^GAPDH^, and relative fold changes were calculated using the 2^−ΔΔCt^ method [52]. Primer sequences used in this study are presented in Table 2.

### 4.5. Immunocytochemistry

Samples were fixed with 4% PFA for 15 min at room temperature, blocked with 1% (*w*/*v*) bovine serum albumin (BSA) in PBS, and permeabilized with 0.1% (*v*/*v*) Triton X-100 in PBS for 1 h at room temperature. Samples were incubated with primary antibody (diluted in 1% (*w*/*v*) BSA in PBS), mouse anti-vimentin (1:200; Santa Cruz Biotechnology, Dallas, TX, USA), overnight at 4 °C. Samples were then washed three times with PBS and incubated with the following secondary antibodies: antirabbit Alexa 555 (1:200; diluted in 1% BSA in PBS), anti-mouse Alexa 555 (1:200; Thermo Fisher Scientific, Waltham, MA, USA), or Alexa Fluor 488 phalloidin (1:200; Thermo Fisher Scientific) for 2 h at room temperature. Nuclei were stained with Hoechst 33342 (2 mg/mL; Thermo Fisher Scientific) for 10 min at room temperature. Fluorescence images were acquired using a confocal microscope (LSM 710; Carl Zeiss, Oberkochen, Germany) at the Soonchunhyang Biomedical Research Core Facility of KBSI.

### 4.6. Image Analysis

For immunofluorescence assays, cells were labeled with vimentin, F-actin and Hoechst 33342 and quantified using MATLAB (MathWorks, Natick, MA, USA). Briefly, the minimum pixel values of the images were subtracted as background noise. Individual cell and nucleus boundaries were determined from F-actin and Hoechst 33342 images using the region of interest and Otsu’s thresholding module. Mean intensity values of the expression of proteins of interest were quantified within these boundaries. The graphic representation of image analyses is presented in Supplementary Figures S3 and S7. Additionally, morphological changes in cell shape were investigated by calculating the cell-shape index as cell circularity (4π × area/perimeter^2^), as previously described [53,54].

### 4.7. Cellular Motility

To analyze the cellular mobility of fibroblasts from all three groups, a wound-healing assay was performed [55]. Cells were seeded onto six-well plates and allowed to grow until they became fully confluent, after which a scratched straight line was created using a 1-mL pipette tip to generate wound gaps, with cell debris gently washed away with PBS. During a 48 h incubation, the wound gaps were measured daily until the gaps were filled. The wound-coverage percentage was calculated [vacant area at day 1 (or day 2)/initially scratched area at day 0] and assessed using ImageJ software (National Institutes of Health, Bethesda, MD, USA).

### 4.8. Cell Proliferation Assay

To examine the cell cycle of dermal fibroblasts, cells were stained and analyzed using the Click-iT EdU Alexa Fluor 488 imaging kit (Thermo Fisher Scientific). Prior to the EdU assay, all three groups of dermal fibroblasts were cultured in serum-free media overnight for cell-growth synchronization followed by incubation in EdU-containing growth medium for 3 h. The EdU assay was performed according to manufacturer instructions to indicate cells in S phase, followed by flow cytometry (Canto FACS Canto II; BD Biosciences) and immunofluorescence analyses at the Soonchunhyang Biomedical Research Core-Facility of KBSI.

### 4.9. Preparation of PAA Gels

PAA hydrogels with a Young’s modulus of 10.6 kPa [30,35] were prepared on top of glass-bottomed dishes. Briefly, the final concentrations of acrylamide and bis-acrylamide were 10% (*w*/*v*) and 0.1% (*w*/*v*), respectively, and the synthesized gels on top of the glass were 40- to 50-μm thick. Human fibronectin (cat# 356008; Corning, Oneonta, NY, USA) was conjugated with PAA using the bifunctional cross-linker N-sulfosuccinimidyl-6-[4-azido-2-nitrophenylamino] hexanoate (Sulfo-SANPAH) to ensure cell adhesion to the gel. As markers for traction force analysis, a final concentration of 0.01% (*w*/*v*) red fluorescent (excitation/emission = 580/605 nm) polystyrene beads (5 µm in diameter; Invitrogen) were added to the PAA solution before polymerization. The Young’s modulus of the PAA gels was confirmed using a previously described method [56].

### 4.10. Cellular Traction Force Analysis

The cellular traction force was calculated as previously reported [22,24]. Briefly, prior to cell-seeding onto PAA gels, NHK fibroblasts were labeled with CellTracker Green CMFDA fluorescent probes (1:500; cat# C2925; Thermo Fisher Scientific), and cells were grown on a fibronectin-conjugated (10 µg/mL) PAA hydrogel in growth medium overnight to allow attachment of seeded cells to the substrate. Images of the fluorescent beads (excitation/emission = 580/605 nm) and cells were acquired in fluorescence and differential interference contrast modes using a laser confocal microscope (LSM 710; Carl Zeiss). A water immersion 40× objective lens (C-Apochromat; NA = 1.2; Carl Zeiss) was used to achieve the appropriate balance between image brightness and field-of-view size. The image pixel size was 0.10 μm/pixel. Displacement of the beads was determined by the particle image velocimetry method coded in MATLAB (MathWorks) by comparing the null-force state (in the absence of cells) and forced state (in the presence of cells) in the images. The window size was 32 × 32 pixels in the X and Y directions, resulting in a spatial resolution of 16 pixels (or 1.6 μm). The lateral stress exerted by the cells was determined from substrate deformation and equilibrium equations for the elastic substrate, as described previously [24]. The partial differential equations were solved with the finite element method (FEM) using commercially available software (Abaqus; Dassault Systèmes, Vélizy-Villacoublay, France). The cellular traction stress was determined from the stress tensor acquired from FEM analysis.

### 4.11. Statistical Analysis

All values are shown as the mean ± standard error of mean of at least three to seven biological replicates for each group, and statistical significance was assessed by one-way analysis of variance (ANOVA) with Tukey’s multiple comparison test using GraphPad Prism software (* *p* < 0.05; ** *p* < 0.01; *** *p* < 0.001 of six or seven biological replicates).

## Figures and Tables

**Table 1:** Categorization of Normal, Hypertrophic and Keloid (NHK) scar tissues.

Variable	Normal Skin Tissue	Hypertrophic Scar	Keloid Scar	Comparison (* *p*)
	(*n* = 6)	(*n* = 7)	(*n* = 7)	
**Age (years)**	47	31	34	0.0805
**Gender**				
Male	6	5	7	
Female	0	2	0	
**Location of keloid**				
Ear	0	3	1	
Extremity	0	0	2	
Trunk	6	4	4	
**Ethnicity**	Asian	Asian	Asian	
**Vancouver Scar Scale**				
Vascularity	0	0.71 ± 1.11	2.29 ± 0.76	0.017
Pigmentation	0	1.14 ± 1.07	1.57 ± 0.98	0.535
Pliability	0	2.43 ± 0.079	4.14 ± 1.21	0.017
Height	0	2.00 ± 0.82	2.16 ± 0.69	0.805
**Total**	0	6.29 ± 1.704	10.00 ± 2.08	0.007

Data represent the mean ± standard deviation for age and frequency (%) for categorical variables. * *p* values were calculated from independent *t*-test for age and chi-squared test or Fisher’s exact test for categorical variables. All skin samples analyzed and used to isolate dermal fibroblasts in this study were from Asian ethnic background.

**Table 2:** List of primers used for qPCR.

Gene	Primer Sequence (5′ to 3′)
***GAPDH***	F-CAC TCC ACC TTT GAC GC
	R-GGT CCA GGG GTC TTA CTC C
***Col-1***	F-CAA GAC AGT GAT TGA ATA CAA AAC CA
	R-GGT CCA GGG GTC TTA CTC C
***FSP-1***	F-TCT TTC TTG GTT TGA TCC TGA CT
	R-AGT TCT GAC TTG TTG AGC TTG A
***α-SMA***	F-AAG CAC AGA GCA AAA GAG GAA T
	R-ATG TCG TCC CAG TTG GTG AT
***Vimentin***	F-AAT CCA AGT TTG CTG ACC TCT CTG A
	R-ACT GCA CCT GTC TCC GGT ACT C
***MMP-1***	F-GGG GCT TTG ATG TAC CCT AGC
	R-TGT CAC ACG CTT TTG GGG TTT
***MMP-2***	F-GAT ACC CCT TTG ACG GTA AGG A
	R-CCT TCT CCC AAG GTC CAT AGC
***MMP-3***	F-CTG GAC TCC GAC ACT CTG GA
	R-CAG GAA AGG TTC TGA AGT GAC C
***MMP-9***	F-GGG ACG CAG ACA TCG TCA TC
	R-TCG TCA TCG TCG AAA TGG GC
***LOX***	F-TTC CAG TAC GGT CTC CCA GA
	R-TGG CCA GAC AGT TTT CCT CC
***LOXL-1***	F-GAG GCC ACC GAC TAC GAT GT
	R-CTG TGG TAA TGC TGG TGG CAG
***LOXL-2***	F-GTA CAA GCC AGA GCA ACC CC
	R-CCT GTG CAC TGG ATC TCG TT
***LOXL-3***	F-AAG CAA CAA CAG TCG AAG CC
	R-TCC AGA GCA GCG AAC TTC AC

## Data Availability

The data presented in this study are available on request from the corresponding authors.

## Supplementary Materials

Supplementary Materials can be found at https://www.mdpi.com/1422-0067/22/4/2198/s1.

## Abbreviations

AFM	atomic force microscopy
ANOVA	analysis of variance
α-SMA	alpha smooth muscle actin
BSA	bovine serum albumin
CI	confidence interval
Col	collagen
CTGF	connective tissue growth factor
DIC	differential interference contrast
DMEM	Dulbecco’s modified eagle’s medium
ECM	extracellular matrix
EDU	5-ethynyl-2′-deoxyuridine
EGF	epidermal growth factor
FBS	fetal bovine serum
FEM	finite element method
FSP-1	fibroblast specific protein 1
GAPDH	glyceraldehyde-3-phosphate dehydrogenase
H&E	hematoxylin and eosin
IPF	idiopathic pulmonary fibrosis
LOX	lysyl oxidase
LOXL	lysyl oxidase-like
MMP	matrix metalloproteinase
PAA	polyacrylamide
PBS	phosphate buffered saline
PFA	paraformaldehyde
PIV	particle image velocimetry
SDS	sodium dodecyl sulfate
Sulfo-SANPAH	N-sulfosuccinimidyl-6-[4′-azido-2′-nitrophenylamino] hexanoate
TFM	traction force microscopy
TGF-β1	transforming growth factor beta 1
TBS	tris-buffered saline
VSS	Vancouver scar scale
