# Supplementary material for: Ameliorating Fibrotic Phenotypes of Keloid Dermal Fibroblasts through an Epidermal Growth Factor-Mediated Extracellular Matrix Remodeling
Source: Int J Mol Sci. 2021 Feb 23;22(4):2198. doi: 10.3390/ijms22042198 (PMC7926382; doi:10.3390/ijms22042198)
Supplement: Supplementary file 1 [file ijms-22-02198-s001.pdf]

Ameliorating Fibrotic Phenotypes of Keloid Dermal Fibroblasts through an Epidermal Growth Factor-Mediated Extracellular Matrix Remodeling

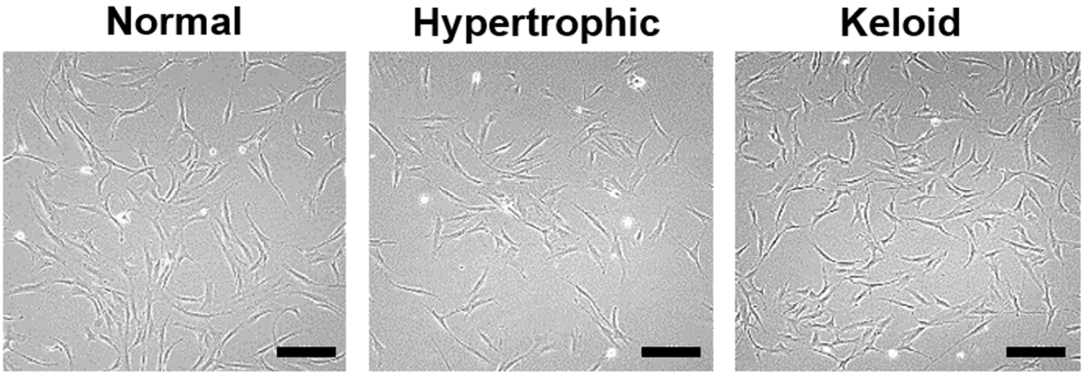

Figure S1: Isolation of primary dermal fibroblasts from NHK patient-derived scar tissues and their cell morphology. Scale bar = 100 μm.

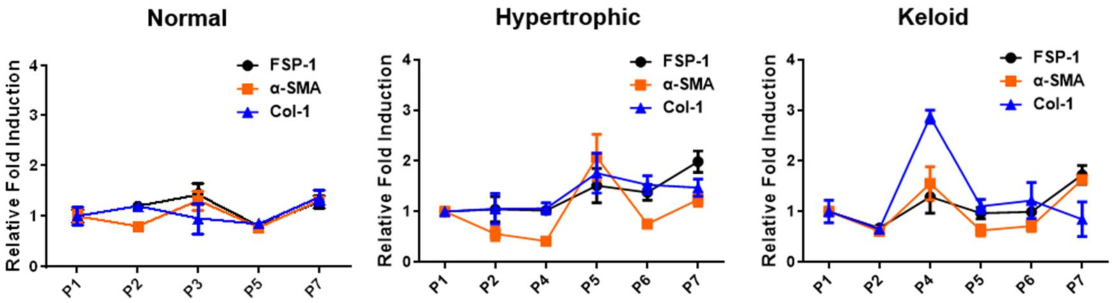

Figure S2: qPCR of the skin fibrosis-/myofibroblast activation-associated markers *FSP-1*, *α-SMA*, and *Col-1* in NHK fibroblasts up to passage 7.

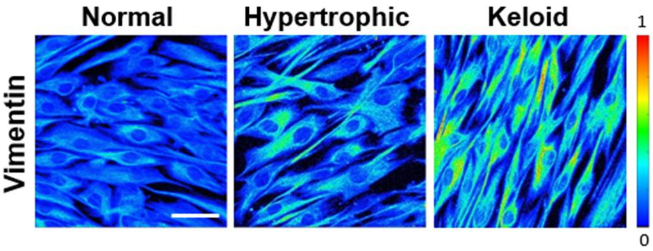

Figure S3: Measurement of the intensity of immunofluorescence staining of vimentin in NHK dermal fibroblasts, quantified by MATLAB (MathWork). The graphic representation of image intensity shows from zero to one intensity ratio. Zero is the lowest and one is the highest values. Scale bar = 50 μm.

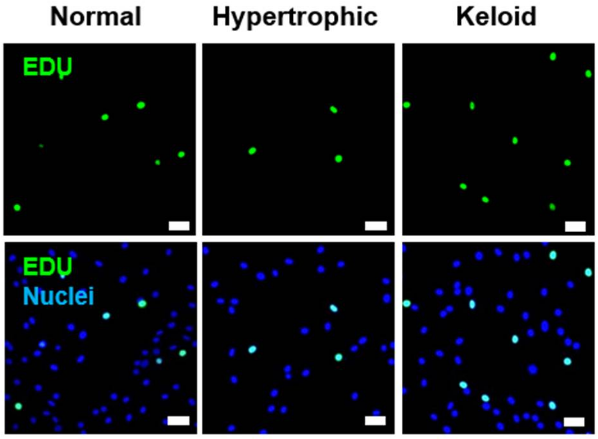

Figure S4: EdU staining of NHK dermal fibroblasts [EdU (green) and Hoechst 33342 (blue)]. Scale bar = 50 μm.

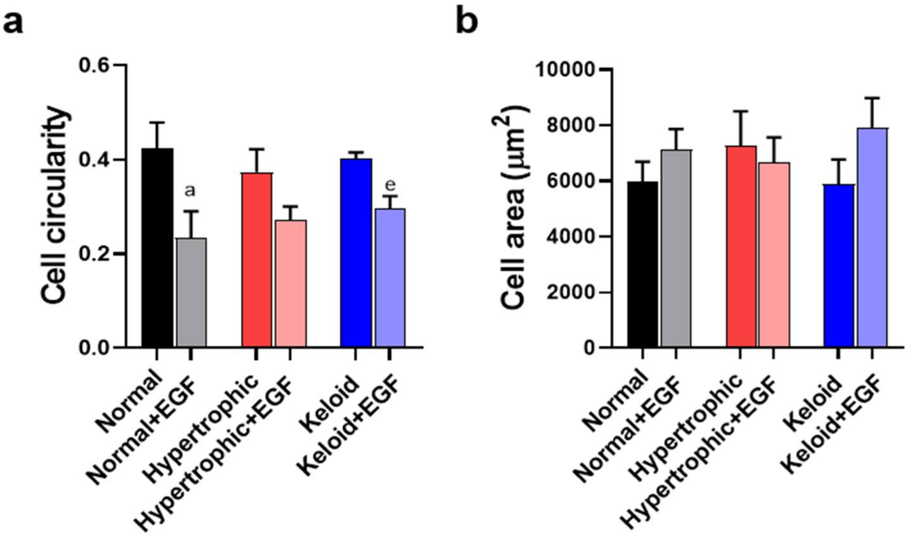

Figure S5: Cell-shape indices, including (a) cell circularity and (b) cell area, for NHK dermal fibroblasts in the absence and presence of EGF treatment. Data are shown as mean ± SEM. [a] $p < 0.05$ vs. normal fibroblast without EGF treatment; and [e] $p < 0.01$ vs. untreated control.

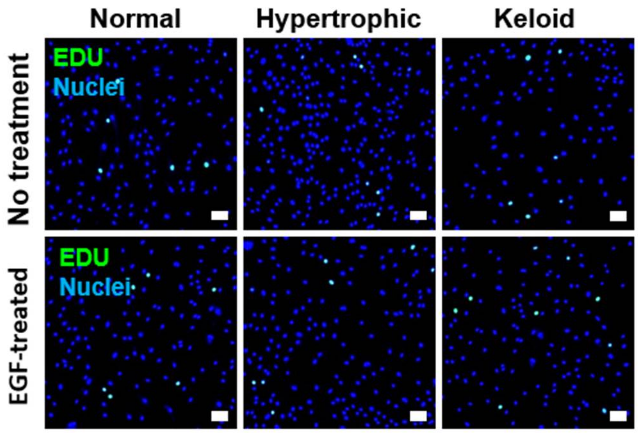

Figure S6: EdU staining of NHK dermal fibroblasts in the presence or absence of EGF treatment [EdU (green) and Hoechst 33342 (blue)]. Scale bar = 50 μm.

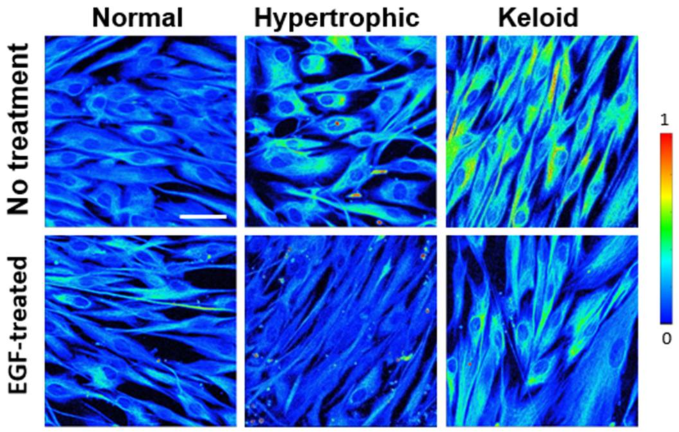

Figure S7: Measurement of the intensity of immunofluorescence staining of vimentin in NHK dermal fibroblasts following EGF treatment quantified by MATLAB (MathWork). The graphic representation of image intensity shows from zero to one intensity ratio. Zero is the lowest and one is the highest values. Scale bar = 50 μm.
